# Verrucous tungiasis - image and clinical findings in a patient with an exuberant case

**DOI:** 10.1016/j.bjid.2023.102772

**Published:** 2023-04-26

**Authors:** Renata Freire Luna, Adryadne da Silva Adolfs, Virginia Vilasboas Figueiras, Marilaine Martins, Luciana Mendes dos Santos

**Affiliations:** a Fundação de Medicina Tropical Dr. Heitor Vieira Dourado (FMT-HVD), Manaus, AM, Brasil; b Universidade Federal do Amazonas (UFAM), Manaus, AM, Brasil

A 28-year-old male patient with low socioeconomic status, living on the street, was brought to the dermatology reference hospital because of exuberant, widespread skin lesions (Fig. 1). He presented a history of untreated mental illness, which made the interview/anamnesis difficult. On examination, plaques of the keratotic surface with blackened crusted areas were observed on several body regions, mainly on the feet (Fig. 2), hands, elbows, knees, and gluteal area. Upon inspection of the lesions, it was possible to observe confluent yellowish papules with a keratotic halo and a blackened central point (Fig. 3).

**Fig. 1:**
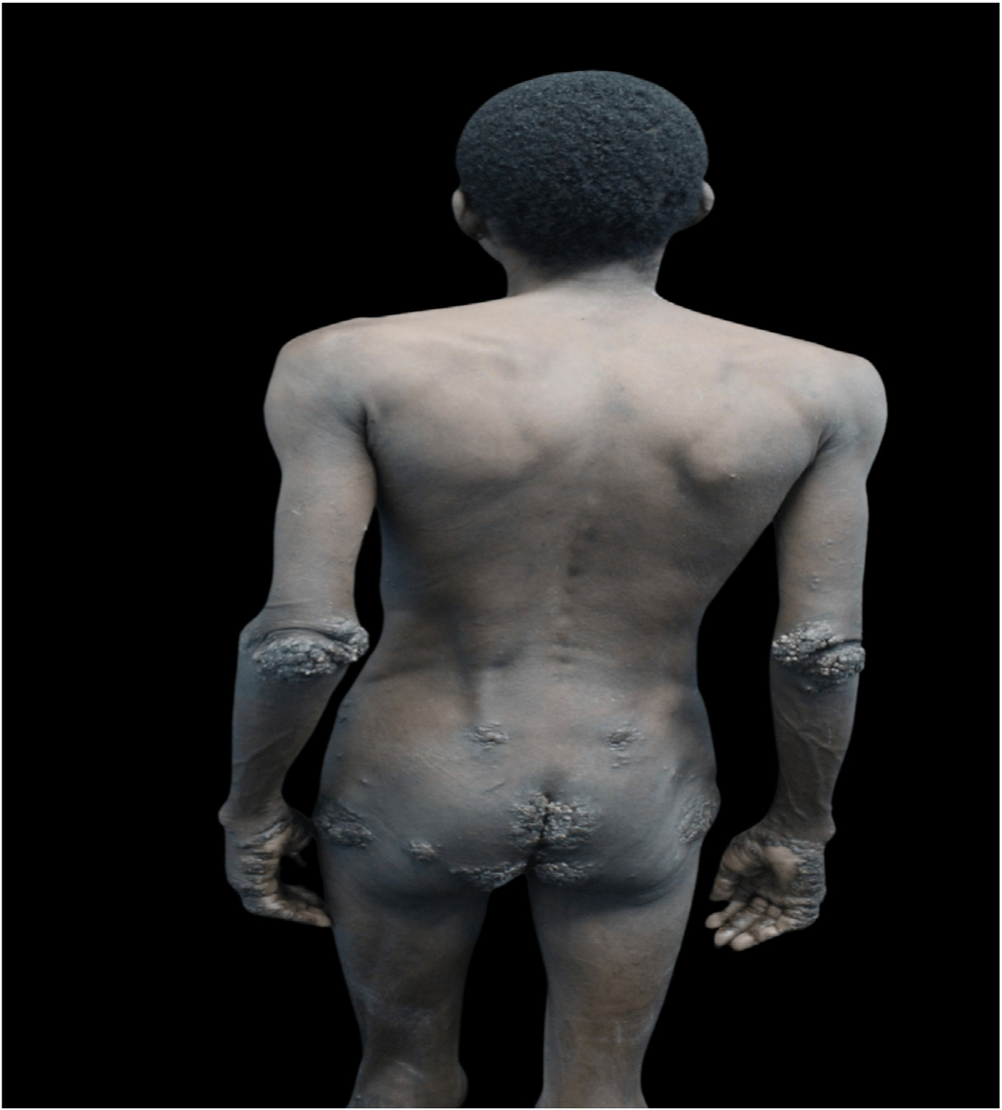
The Figure show hyperkeratotic lesions observed on the elbows, lower back, hips, and hands.

**Fig. 2:**
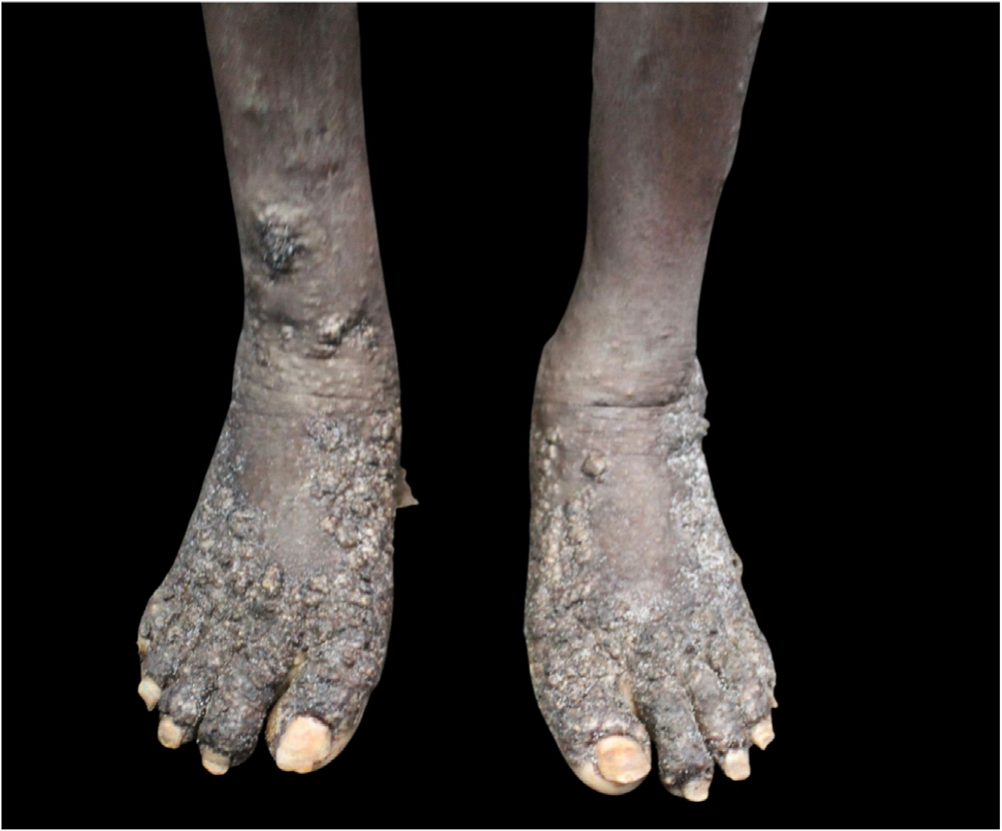
The Figure show hyperkeratotic plaques, forming a verrucous surface on the patient feet.

**Fig. 3:**
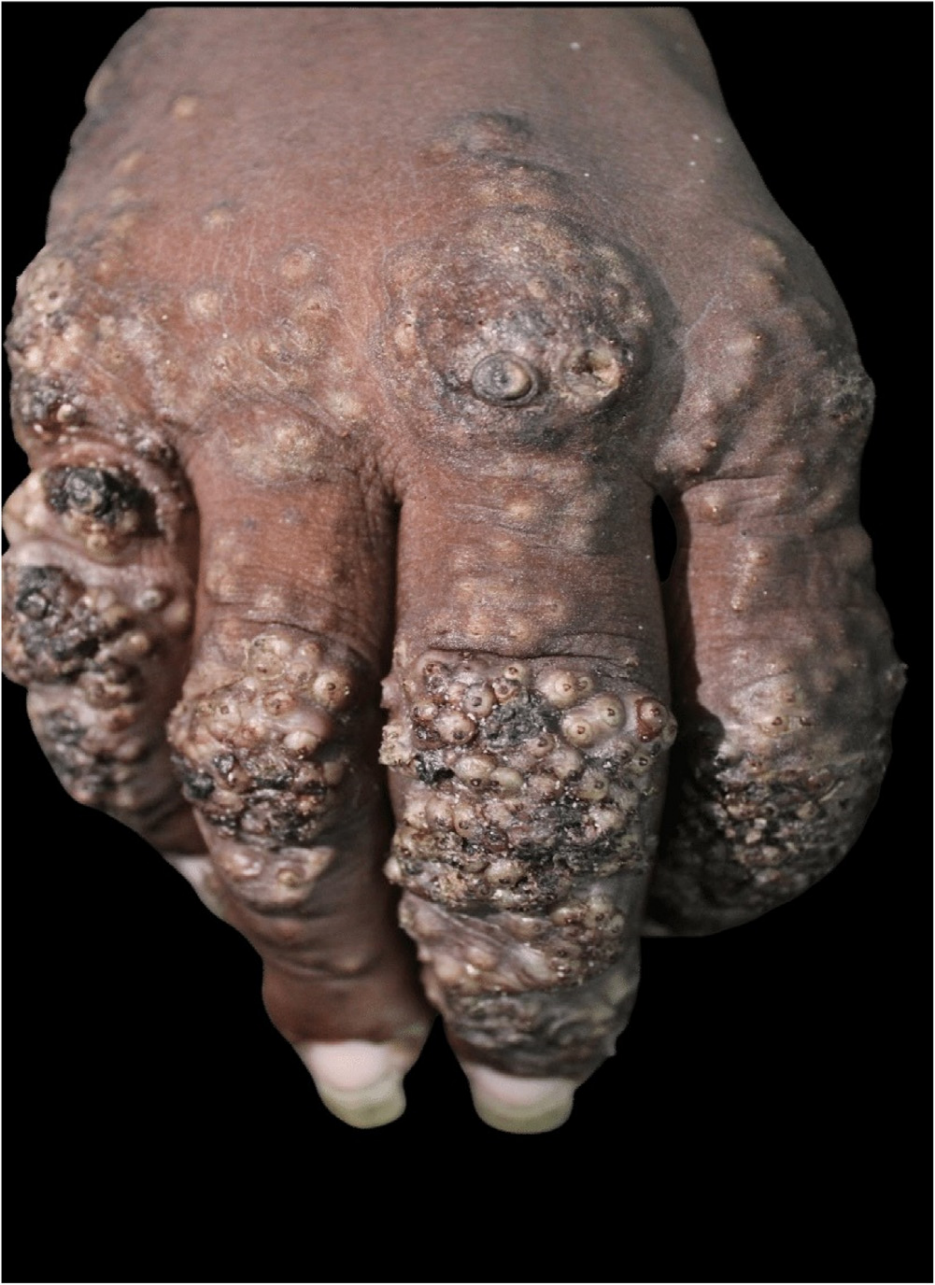
The Figure show the confluent yellowish papules with a keratotic halo and a blackened central spot on the patient hand.

The dermatoscopic examination showed a yellowish structure surrounded by a whitish halo and a brown central spot^1^ (Fig. 4). The clinical diagnosis of Tungiasis was complemented by the microscopic finding of the entire cycle of the disease, through a fragment of skin, which confirmed the presence of the Tunga penetrans flea, her eggs, and the larval stage, as shown in Fig. 5, Fig. 6, Fig. 7.

**Fig. 4:**
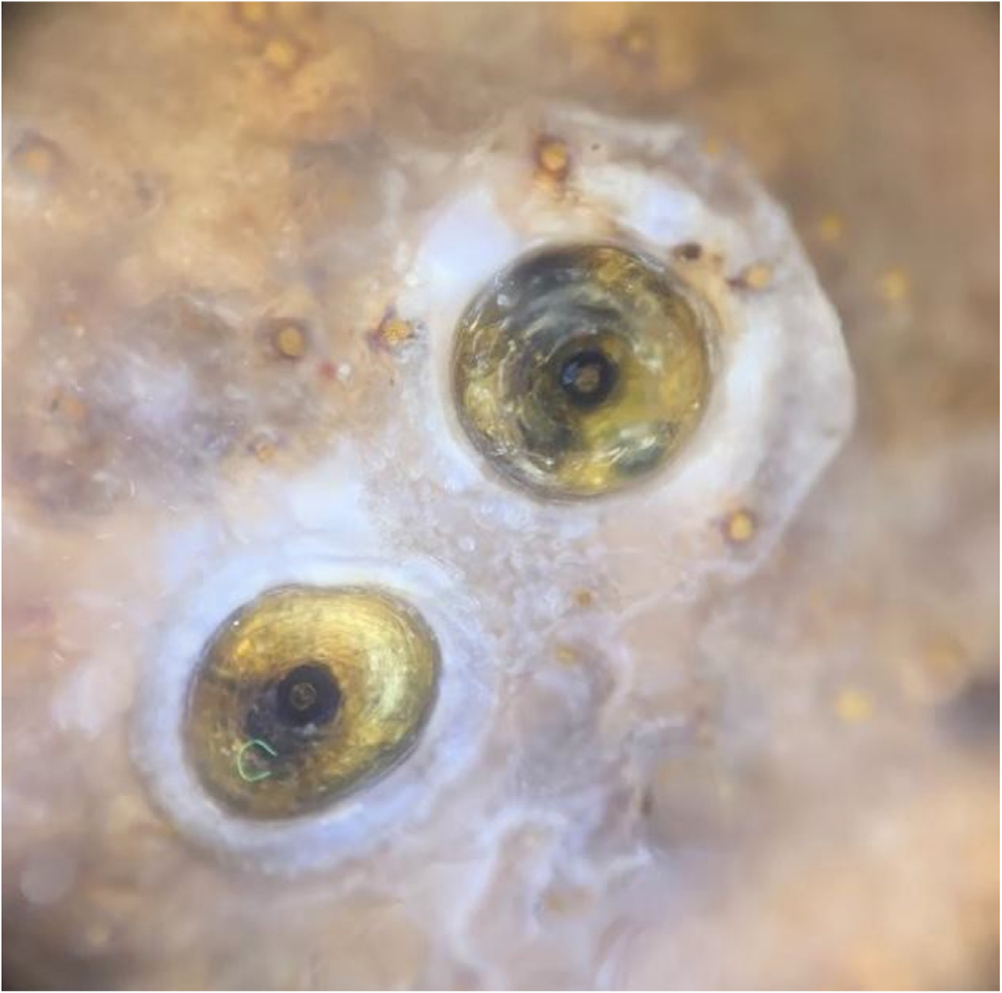
The Figure shows the Dermatoscopy magnified 10 times, displaying yellowish structures surrounded by a whitish halo and a brown central spot.

**Fig. 5:**
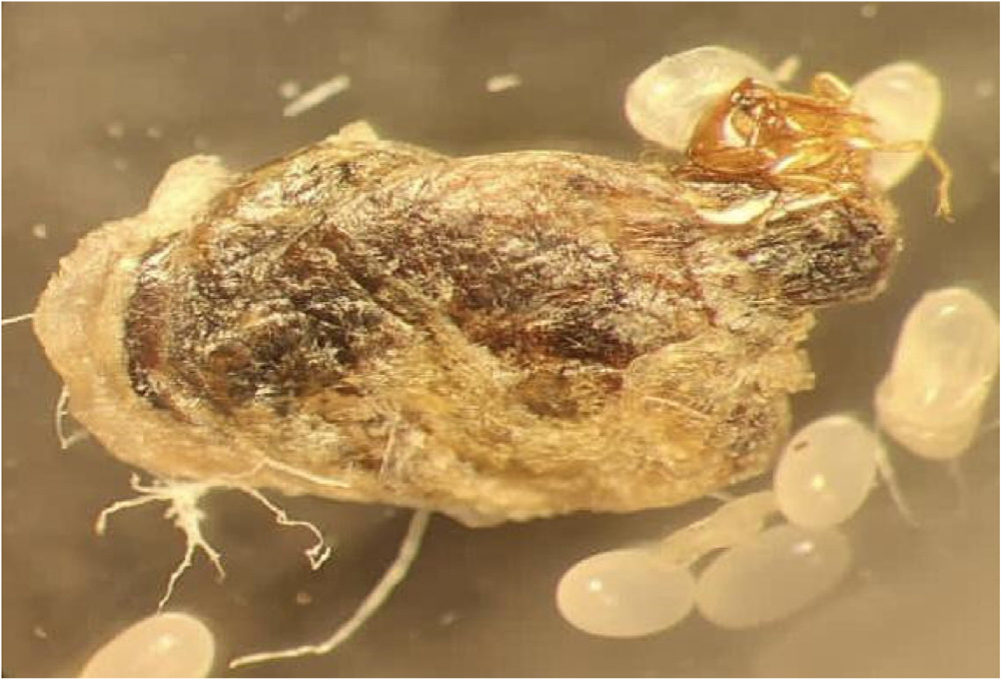
It is observed the optical microscopy magnified 400 times demonstrating the flea tunga penetrans.

**Fig. 6:**
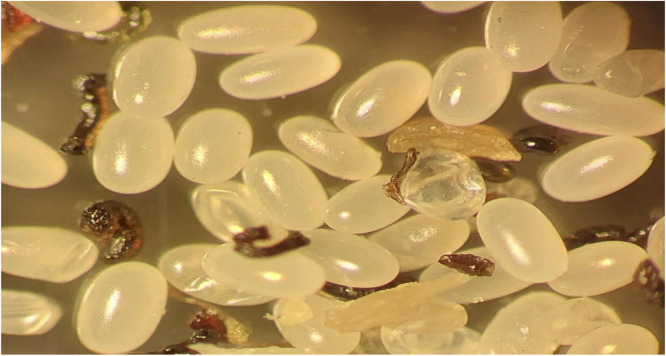
It is observed the optical microscopy magnified 400 times illustrating the eggs of the flea tunga penetrans.

**Fig. 7:**
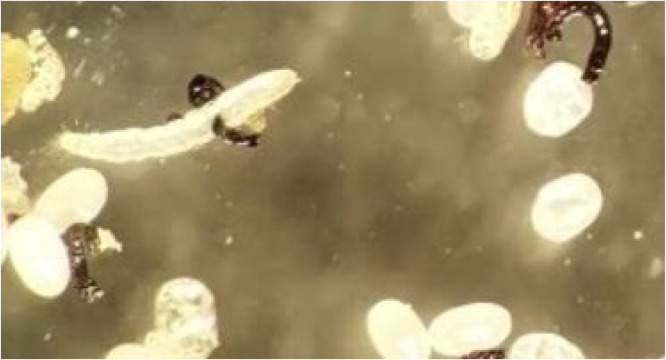
It is observed the optical microscopy magnified 400 times displaying the larval stage in the tungiasis cycle.

Treatment was instituted with ivermectin 6mg (2 tablets/weekly), plus anti-tetanus vaccination, and prescribed vaseline salicylate 20% under occlusion in the lesions with progressive improvement. Tungiasis is a neglected tropical disease with high prevalence and most of the time it has a self-limited course. In endemic environments, reinfection is the usual rule and the parasite load accumulates over time.^2^

Alcoholics, people with mental illnesses, and people living in underdeveloped communities are more likely to develop extensive symptoms. The fleas prefer to penetrate pre-existing lesions. Repeated infections can lead to pseudoepitheliomatous hyperplasia and then could lead to verrucous tungiasis.^3^^,^^4^

The verrucous form is an exuberant clinical manifestation that is not well described and makes a differential diagnosis with other verrucous diseases such as subcutaneous mycoses, warts, and cutaneous tuberculosis. Extensive and untreated cases may progress with bacterial infections, lymphedema, deep ulcers, tetanus, and even self-amputations. And despite the high prevalence, there is still no consensus on the optimal treatment.^2^^,^^4^

## Conflicts of interest

The authors declare no conflicts of interest.
